# Retention and Engagement in Culturally Adapted Digital Mental Health Interventions: Systematic Review of Dropout, Attrition, and Adherence in Non-Western, Educated, Industrialized, Rich, Democratic Settings

**DOI:** 10.2196/80624

**Published:** 2026-01-28

**Authors:** Tanya Tandon, Rajashree Biswas, Quentin Meteier, Karl Daher, Omar Abou Khaled, Björn Meyer, Thomas Berger, Rashmi Gupta, Chantal Martin Soelch

**Affiliations:** 1 Department of Clinical and Health Psychology, University of Fribourg, Rue P.-A.-de-Faucigny 2, Fribourg, 1700, Switzerland, 41 779767637; 2 Cognitive and Behavioural Neuroscience Laboratory, Department of Humanities and Social Sciences, Indian Institute of Technology Bombay, Mumbai, India; 3 HumanTech Institute, HES-SO University of Applied Sciences Western Switzerland, Fribourg, Switzerland; 4 Haute École d’Ingénierie et d’Architecture de Fribourg, Fribourg, Switzerland; 5 Department of Research, Gaia AG, Hamburg, Germany; 6 Institute of Psychology, University of Bern, Bern, Switzerland; 7 Koita Centre for Digital Health, Indian Institute of Technology Bombay, Mumbai, India

**Keywords:** digital mental health, cultural adaptation, non-WEIRD settings, dropout, attrition, adherence, online intervention, cultural tailoring, western, educated, industrialized, rich, democratic

## Abstract

**Background:**

Digital mental health interventions (DMHIs) offer scalable and cost-effective support for mental health but are predominantly developed in WEIRD (western, educated, industrialized, rich, democratic) contexts, raising questions about their global applicability. Dropout, attrition, and adherence rates critically influence DMHI effectiveness yet remain poorly characterized in culturally adapted formats.

**Objective:**

This systematic review aimed to (1) synthesize evidence on dropout, attrition, and adherence in culturally adapted DMHIs delivered to non-WEIRD adult populations and (2) assess the methodological quality of the included studies.

**Methods:**

PsycINFO, PubMed, and ScienceDirect were systematically searched for randomized controlled trials published in English between January 2014 and April 2024. Screening and data extraction followed PRISMA (Preferred Reporting Items for Systematic Reviews and Meta-Analyses) guidelines, and methodological quality was evaluated using the Appraisal Tool for Cross-Sectional Studies tool. Extracted variables included dropout, attrition, adherence, adaptation techniques, and clinical outcomes.

**Results:**

Twenty-three randomized controlled trials (n=4656) from diverse regions met inclusion criteria. Attrition ranged from 5.3% to 87% (median 18.4%), dropout from 0% to 66% (median 18.7%), and adherence from 26.3% to 100% (median 71%). Deep, participatory adaptations—such as language translation combined with culturally resonant content, stakeholder engagement, and iterative refinement—were consistently associated with lower dropout (<11%) and higher adherence (>75%). In contrast, surface-level adaptations (eg, translation only) showed higher dropout (up to 56%). Studies that incorporated both cultural tailoring and human support reported the most favorable engagement and clinical outcomes (eg, reductions in insomnia, depression, and anxiety). Most studies (91%) were rated as “Good” quality, although some lacked representative sampling or objective engagement metrics.

**Conclusions:**

Comprehensive and participatory cultural adaptation is associated with engagement and effectiveness of DMHIs among non-WEIRD populations. Future research should integrate hybrid human-digital delivery models, objective engagement metrics, and larger multicenter trials to improve generalizability and scalability.

## Introduction

Digital mental health interventions (DMHIs) have seen explosive growth in recent years [1], offering scalable, cost-effective ways to broaden access, reduce costs, and empower users to self-manage their well-being [2,3]. These platforms—including mobile apps, video-based therapy, peer-led communities, and interactive web modules—provide flexible, on-demand support [4], with users reporting benefits such as scheduling and location flexibility, low effort, enhanced access and anonymity, greater trustworthiness with facilitators [5]. Pandemic-related demand and advances in digital access have accelerated DMHI development globally [6,7].

Alongside this expansion, longstanding debates about cultural relevance in public health have extended into the digital realm [8,9]. Most digital health research remains rooted in WEIRD (western, educated, industrialized, rich, democratic) settings [10-12], raising questions about generalizability. While some small-scale adaptations—such as a sleep-support app tailored for German refugees—have demonstrated high satisfaction and comparable adherence [13]. However, a systematic review of over 10,000 participants found no consistent efficacy advantage for culturally adapted interventions [14]. These mixed findings suggest that adaptation may boost initial uptake but does not guarantee sustained engagement or better outcomes.

A critical factor underlying these mixed results is participant retention [15]. High dropout and attrition can erode both effectiveness and cost-efficiency [16], making retention metrics essential for evaluation. Research typically focuses on three core measures: the dropout rate (discontinuation before completion [17]), the attrition rate (loss to follow-up or ceased usage [18]), and the adherence rate (completion of prescribed sessions [19]). Understanding what drives these outcomes—be it cultural fit, usability barriers, or motivational factors—is key to crafting sustainable, impactful digital interventions [20,21].

Therefore, the present systematic review aims to comprehensively examine retention and engagement outcomes in culturally adapted DMHIs implemented among non-WEIRD adult populations. Specifically, this review seeks to (1) synthesize evidence on dropout, attrition, and adherence rates across studies and (2) evaluate the methodological quality of the included trials to identify strengths, limitations, and factors associated with higher retention and adherence.

By addressing these objectives, the review intends to generate evidence-based recommendations to guide the design and implementation of culturally responsive DMHIs worldwide.

## Methods

### Article Search and Selection

This review was preregistered on PROSPERO (Prospective Specific Evaluation of Reviews) (CRD42025641863 [22]). The literature search took place from February 2024 and ended in July 2024. To ensure comprehensiveness, we used three search strategies: database searches and manual searches of reference lists of relevant articles.

### Database Search

The review followed the guidelines of the PRISMA (Preferred Reporting Items for Systematic Reviews and Meta-Analyses) [23]. A comprehensive search was conducted in the following electronic databases: (1) PsycINFO, (2) PubMed, and (3) ScienceDirect. The search strategy was designed to identify both quantitative and qualitative studies focusing on culturally adapted mental health interventions delivered via digital platforms (eg, e-mental health, mobile applications). Search terms were developed using combinations of keywords related to cultural adaptation (eg, “culturally appropriate,” “adapted intervention”) and intervention modality (eg, “digital health,” “mobile app,” “e-mental health”). To ensure breadth and sensitivity, the search strategy incorporated a wide range of related terms. The complete search strategy is provided in Multimedia Appendix 1 [24]. Given that previous reviews included studies up to 2014, only studies published between January 2014 and April 2024 were included. The systematic search process and the rationale for study inclusion and exclusion were documented in accordance with PRISMA standards (see Checklist 1). Two lead authors independently reviewed articles for inclusion, with disagreements resolved through discussion and consensus.

### Inclusion and Exclusion Criteria

The population, intervention, control, and outcomes model served as the foundation for the creation of the inclusion criteria [25]. People from non-WEIRD societies were referred to as part of the population [12]. This systematic review focuses on DMHIs adapted for non-WEIRD populations, with clearly defined inclusion and exclusion criteria.

1. Eligible interventions must be internet-, computer-, or mobile-based to address mental health problems, including depression, anxiety, or trauma.
2. They must also be culturally adapted for the target group to align with the population’s cultural context.
3. The target population includes adults aged 18 years or older from non-WEIRD cultural backgrounds that differ from the original intervention target group.
4. Only randomized controlled trials (RCTs) published in peer-reviewed English-language journals within the last 10 years are included, with no restrictions on the type of setting (eg, rural, urban, clinical, or non-clinical).

Exclusion criteria were excluded if they (1) involved interventions that lack cultural adaptation, (2) targeted individuals under 18 years, (3) were nondigital interventions, (4) were observational studies, case reports, and qualitative studies, and (5) were articles not published in English or outside the 10-year timeframe. By adhering to these criteria, the review will evaluate the impact of cultural adaptations on reducing drop-out rates and the overall effectiveness of these interventions.

### Data Screening and Eligibility

After duplicates were removed using EndNote (version 20.3; Clarivate), the remaining records were screened manually using Microsoft Excel. The titles and abstracts were independently screened by two lead authors, based on pre-established inclusion and exclusion criteria. The level of agreement between the screeners was 85% across title/abstract screening, data extraction, and quality assessment stages. Discrepancies were resolved through discussion until consensus was reached.

### Data Extraction

#### Overview

Data extraction was conducted manually using a predesigned Excel spreadsheet. The data extraction plan was developed in accordance with PRISMA guidelines and informed by recent reviews on digital health interventions among minority populations [26-28]. One author extracted the data, and another author independently cross-checked the entries for accuracy. As this is a systematic review, no imputation or sensitivity analyses were conducted. Medians and ranges were calculated only for studies that explicitly reported each outcome, and the number of contributing studies (n) is provided for each summary statistic.

#### Extraction of Participant Demographics

Demographic information, including participants’ age, gender, and cultural background, was extracted directly from the study descriptions or participant tables. Missing or incomplete demographic data were noted in the extraction sheet.

#### Extraction of Recruitment Settings

Recruitment methods and settings (eg, community-based, clinical, or online) were coded from the methods section of each study. When not explicitly stated, the inferred setting was noted.

#### Extraction of Engagement Metrics (Dropout, Adherence, and Attrition)

Engagement data were extracted as follows: dropout was defined as noncompletion of the intervention; attrition as loss to follow-up; and adherence as the proportion of sessions completed. If data were not reported, this was recorded as “not available.”

#### Extraction of Cultural Adaptation Strategies

Details on cultural adaptation (eg, translation, content tailoring, stakeholder involvement, and iterative feedback) were extracted from intervention descriptions. Adaptations were coded as surface-level or deep-level.

#### Extraction of Clinical Outcomes

Primary and secondary clinical outcomes (eg, depression, anxiety, insomnia) and their corresponding measurement tools were extracted and coded for direction of effect (improvement, no change, or worsening).

### Quality Assessment

Two authors independently conducted the quality assessment of all included quantitative studies using the Appraisal Tool for Cross-Sectional Studies [29]. Disagreements were resolved by discussion. Each item was rated as “yes,” “no,” or “do not know,” with scores assigned according to conventions used in previous reviews [30,31]: yes or not applicable (N/A)=1 point; no or do not know=0 points. Total scores ranged from 0 to 20, with studies rated as good (≥15), fair (10-14), or poor (<10).

## Results

### Study Selection

**Figure 1.**
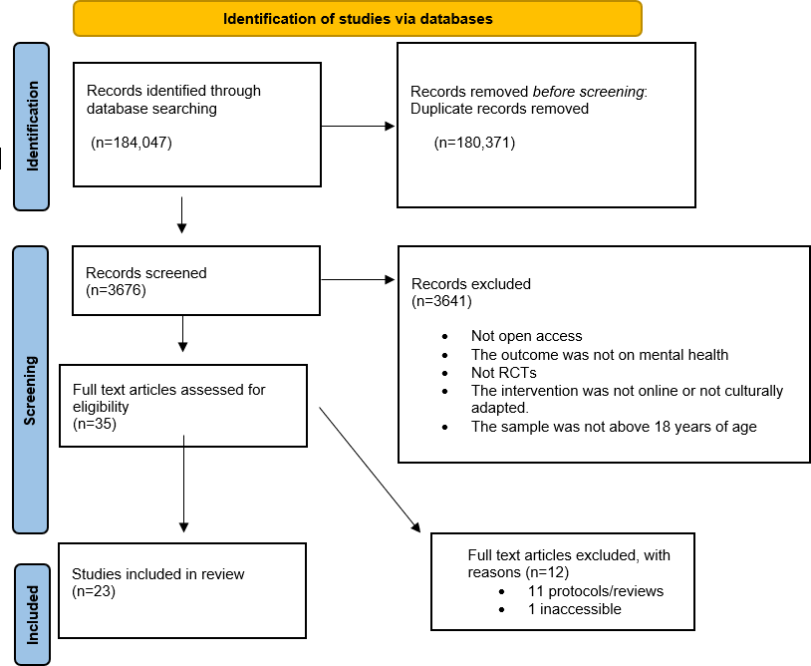
PRISMA flowchart. RCT: randomized controlled trial.

A total of 184,047 records were identified through the database search. After removing duplicates (n=180,371), 3676 articles remained for title and abstract screening. Of these, 3641 were excluded based on the predefined inclusion and exclusion criteria. The remaining 35 articles were assessed for eligibility by the authors. Eleven studies were excluded at this stage because they were study protocols or review articles, and one study met all inclusion criteria but was excluded from the final review due to inaccessibility; attempts to obtain the full text through institutional subscriptions and direct author contact were unsuccessful. Ultimately, 23 articles were included in the final review. The study selection process is summarized in Figure 1, and detailed study descriptions are available in Multimedia Appendix 2 and Table 1.

**Table 1.** Study description of the selected studies (n=23).

Author and year	Country of origin	Adapted country	Recruitment settings	Age (y), mean (SD)	Demographics	Intervention type	Platform used	Adaptation framework used	Dropout rate (%)	Attrition rate (%)	Adherence rate (%)	Primary outcome measure
Zhang et al [32] (2023)	China	China	Not reported	49.67 (14.49)	Insomnia (chronic insomnia disorder), Chinese; 74.4% female	DCBT-I^a^ app	Smartphone-based app	Not reported	11	11	94	Insomnia (ISI^b^)
Spanhel et al [13] (2022)	Germany	Germany	Online	26.8 (4.4)	International students in Germany (92.6% with insomnia); 49.4% female	StudiCare Sleep-e based on CBT^c^	Web-based intervention on Minddistrict platform	Adaptation included content (eg, removal of sleep restriction), duration (shortened from 6 to 3 modules), language (translation into English), and the use of students as case examples.	56	46	44	Insomnia (ISI)
Zeng et al [33] (2020)	China	China	Outpatient clinics; online	28 (5.8)	HIV seropositive individuals with depressive symptoms; 5.33% female.	WeChat-based mHealth^d^ intervention	WeChat-based (app-based)	Not reported	50	8	100	Depression (CES-D)^e^
Guo et al [34] (2022)	China	China	Outpatient clinics; online	28.3 (5.85)	HIV seropositive individuals with depressive symptoms; 7.65% female.	Run4Love based on CBSM^f^	WeChat with multimedia materials, automated tracking, and phone check-ins.	CBSM adapted in Chinese context	41	41	50	Depression (CES-D)^e^
Campbell et al [35] (2023)	United States	United States	Outpatient clinics	38. 6 (10.3)	American Indian and Alaska Native in the United States; 45.3% female, 1.9% transgender.	TES-NAV^g^	Smartphone app or clinic tablets	Integrated multiframework adaptation: (1) Ecological Validity Model (Bernal et al [36], 2009) to align language, persons, metaphors, content, concepts, methods, goals, and context; (2) Barrera et al’s [ 37] (2013) 5-step systematic cycle (information gathering → preliminary design → pilot test → refinement → final trial); and (3) Wingood and DiClemente’s [38] (2008) cultural-tailoring principles. All steps were iteratively co-designed with four native clinicians/psychologists, individuals with lived experience, and community reviewers.	49	31	74	Abstinence from heavy drinking or drug use (urine screen and self-report)
Lindegaard et al [39] (2019)	Sweden	Sweden	Not reported	33.86 (8.2)	Depressive Kurdish people of Sweden; 46% female	ICBT^h^	Secure online platform: Iterapi	No formal framework cited	28	44	52	Depression (BDI-II)^i^
Silva et al [40] (2020)	United States	United States (Spanish speaking population)	Not reported	42.7 (11.6)	Native Spanish speaking individuals; DSM^j^ IV abuse and substance dependence; 32.6% female	CBT4CBT^k^	Web based	Cultural constructs by Anez et al [41,42] (2005, 2008)	12	5	88	Change in SUD^l^ (ASI^m^)
Yeung et al [43] (2016)	United States	United States (Chinese American immigrants)	Online	50 (14.5)	Monolingual Chinese Americans with depression, 63% female	T-CSCT^n^	Polycom VSX3000 systems were used for videoconferencing. Later, switched to Skype	Culturally sensitive psychiatric consultation using the Engagement Interview Protocol (EIP).	Not stated	Not stated	Not stated	Depression (HDRS17)^o^
Sarfraz et al [44] (2023)	Pakistan	Pakistan	Online	22.90 (3.57)	Undergraduate and postgraduate university students, 69% female	MTC^p^	Zoom and email	Medical Research Council (MRC) guidelines for complex interventions; Heuristic framework for cultural adaptation	28	28	48	Psychological distress (CORE-OM)^q^; psychological well-being (PWB-S)^r^; dispositional mindfulness (FFMQ^s^)
Zemestani and Fazeli Nikoo [45] (2020)	Iran	Iran	Not reported	29.59 (3.59)	Pregnant women (1‐6 wk of gestational age)	MBCT^t^	In-person group sessions + audio for home practice (offline)	No formal framework cited	13	23	87	Depression (BDI-II); Anxiety (BAI)^u^; emotional regulation (ERQ)^v^; well-being (SPWB)^w^
Spruill et al [46] (2021)	United States	United States	Outpatient clinics	43.3 (11.3)	Hispanic ethnicity; 67% primary Spanish speaker; 71% female	Project UPLIFT^x^, adapted from MBCT	Telephone	No formal framework cited—adaptations informed by qualitative research and best practices (eg, focus groups, simplification, cultural tailoring);	14	7	75	Depression (PHQ-9)^y^
Zhang et al [47] (2023)	China	China	Outpatient clinics; online	30.29 (4.29)	Pregnant women in China	GSH-MBI^z^	WeChat mini-program	No formal framework cited—adaptations relied on culturally tailored content delivered through WeChat.	19	16	81	Depression (EPDS)^aa^; Anxiety (GAD-7)^ab^
Benjet et al (2023) [48]	Mexico and Colombia	Mexico and Colombia	Online	21.4 (3.2)	University students; 1038 women (78.7%); 725 participants (55.0%) came from Mexico	i-CBT^ac^	Web based	Iterative user-centered model	Not reported	32	Not reported	Anxiety (GAD-7) and depression (PHQ-9) scores
Vaca et al [49] (2023)	United States	US Latino adults	Not reported	36.2 (11.2)	433 (51.5%) were male, 407 (48.5%) were female and 83% of them were from Puerto Rico	AB-CASI^ad^	Computer tablets (iPad 4th Generation; Apple Inc)	Not explicitly named	Not reported	24	Not reported	Alcohol Use Disorders (AUDIT)^ae^
Zhou et al [50] (2022)	United States	United States (specific adaptation for Black women in the United States)	Not reported	59.5 (8)	American Black women	SHUTi-BWHS^af^ based on CBT-I^ag^	Web-based	Stakeholder-informed, iterative cultural adaptation process (not explicitly a formal framework, but uses participatory design principles)	22	16	78	Insomnia (ISI)
Javier et al [51] (2025)	United States.	Filipino families	Not reported	42 (5.6)	Filipino; parents: 81.7% females, 16.3% males	Incredible Years School Age Basic and Advance Programs	Web based	Language, persons, metaphors, content, concepts, goals, methods, and context based on Bernal et al, [52] (1995) framework including language, persons, metaphors, content, concepts, goals, methods, context	18	18	Not explicitly mentioned	Parenting practices (PPI)^ah^; Parenting stress (PSI)^ai^; Child’s behavior (CBCL)^aj^; Child-reported anxiety and depression symptoms (SCARED^ak^ and CDI^al^)
Owen et al [53] (2022)	United States	African Americans in the United States	Outpatient clinics; online	65.9	African Americans; 14 females and 3 males	CBT	In-person and online group formats	Agricultural Coping Model using amalgam of norms from West Africa, cultural traditions and practices from European/American society, and experiences of historical and contemporary racism in the United States	Not reported	18	Not reported	Cognitive function (MoCA)^am^
Lindegaard et al [54] (2021)	Sweden	Sweden (for Arabic-speaking immigrants and refugees)	Online	37.5 (11.4)	Arabic-speaking population, 25 females and 34 males	ICBT	Web-based with asynchronous therapist messaging and feedback	Iterative adaptation and tailoring process (focus groups + pilot feedback)	39	Not reported	Not reported	Depression (PHQ-9)
Yamaguchi et al [55] (2019)	Japan	Japan	Community	20.25 (1.31)	University students, 26 females and 70 males	FSC^an^; IBSS^ao^	In-person (initial session)+ email follow-up	Not reported	Not reported	28	Not reported	Reported behavior, and the other on intended behavior (RIBS-J)^ap^; Mental Illness and Disorder Understanding (MIDUS)^aq^
Ellis et al [56] (2022)	United States	Egypt	Not reported	Range 20‐54 (28)	Arabic speaking population, 62 females and 25 males	CBT-based PTSD^au^ for online coaching in Arabic	Web based	Bernal et al (1995) [52] framework	Not reported	13	Not reported	PTSD (PCL-5)^ar^
Sun et al [57] (2022)	United States	China	Online	22.21 (2.67)	University students, 73.7% females	Mindfulness-based mHealth	Web-based via WeChat (mini-program) and Zoom	Informed by focus group input; rapid iterative tailoring (no formal framework named)	9	13	Not reported	Anxiety (GAD-7); depression (PHQ-9)
Jacobs et al [58] (2016)	United States	Ecuador	Community	≥18	Students	Familias Unidas is a parent-centered intervention	In-person sessions; group and family-based, Audio Computer-Assisted Self-Interviewing	Barrera et al’s [59] (2017) surface-structure adaptation model—constructs vetted against Ecuadorian family norms/laws; parent review for linguistic clarity; minor wording and local prevalence data updates; original Hispanic-acted skill videos retained, with no deep-structure changes required	Not reported	Not reported	Not reported	Drug use (self-reported by adolescents); Adolescent sexual behavior, drug use, and violence (ACASI)^as^
Barrera et al [60] (2015)	United States	Spain	Online	30.19 (5.57)	Pregnant women, majority resided in Chile, Spain, Argentina, Mexico, Colombia, and the United States. Most were Spanish speaking (82.9%) of Latino/Hispanic ethnic identity (71.3%), and identified their racial background as Caucasian/European (53.2%) or Mestizo (31.8%)	Mothers and Babies Internet Course/Curso Internet de Mamás y Bebés (e-MB) based on CBT approach	Web-based accessed via email login links	Iterative user-feedback model (usability testing, linguistic translation, visual editing)				

a DCBT-I: digital cognitive behavioral therapy for insomnia.

b ISI: Insomnia Severity Index.

c CBT: cognitive behavioral therapy.

d mHealth: mobile health.

e CES-D: Center for Epidemiological Studies Depression Scale.

f CBSM: cognitive behavioral stress management.

g TES-NAV: therapeutic education system-native version.

h ICBT: inference-based cognitive behavioral therapy.

i BDI-II: Beck Depression Inventory-II.

j *DSM*: *Diagnostic and Statistical Manual*.

k CBT4CBT: Web-based cognitive behavioral therapy program.

l SUD: substance use disorder.

m ASI: Addiction Severity Index.

n T-CSCT: telepsychiatry-based culturally sensitive collaborative treatment.

o HDRS17: Hamilton Depression Rating Scale.

p MTC: online mindfulness training course.

q CORE-OM: clinical outcomes routine evaluation-outcome measure.

r PWB-S: Ryff’s psychological well-being scale.

s FFMQ: Five-Facet Mindfulness Questionnaires.

t MBCT: mindfulness-based cognitive therapy.

u BAI: Beck Anxiety Inventory.

v ERQ: Emotion Regulation Questionnaire.

w SPWB: scales of psychological well-being.

x UPLIFT: using practice and learning to increase favorable thoughts.

y PHQ-9: Patient Health Questionnaire.

z GSH-MBI: digital guided self-help mindfulness-based intervention.

aa EPDS: Edinburgh Postnatal Depression Scale.

ab GAD-7: Generalized Anxiety Disorder.

ac i-CBT: Internet-delivered cognitive behavioral therapy.

ad AB-CASI: automated bilingual computerized alcohol screening and intervention.

ae AUDIT: Alcohol Use Disorders Identification Test.

af SHUTi-BWHS: tailored version of automated internet-delivered treatment called Sleep Healthy Using the Internet for Black women.

ag CBT-I: cognitive behavioral therapy for insomnia.

ah PPI: Parenting Practices Interview.

ai PSI: Parenting Stress Index.

aj CBCL: child behavior checklist.

ak SCARED: parent's screening for child anxiety-related disorders.

al CDI 2: parent and child report Children’s Depression Inventory 2.

am MoCA: Montreal cognitive assessment.

an FSC: filmed social contact.

ao IBSS: internet-based self-study.

ap RIBS-J: reported and intended behavior scale – Japanese version.

aq MIDUS: mental illness and disorder understanding scale.

ar PCL-5: post-traumatic stress disorder checklist.

as ACASI: adolescent sexual behavior, drug use, and violence.

at MDE: Major Depressive Episode Screener—current/lifetime version.

au PTSD: post-traumatic stress disorder.

### Participant Demographics

Participants (n=4656; see Multimedia Appendix 2) represented diverse cultural and demographic backgrounds. Several studies focused on specific subpopulations like pregnant women who were targeted in two studies [47,60,61], while young adults aged 18 to 30 years were the focus of 39% (8/23) [33,34,44,48,55,57,61,62]. Chinese participants were the most frequently represented cultural group, included in 26% (6/23) of studies [32-34,43,47,57]. University students from various countries were the focus in 21% (5/23) of studies [44,48,55,57,62]. Studies involving Middle Eastern or Arabic-speaking populations accounted for 17% (4/23) [13,54,56,61]. A significant proportion of studies examined Hispanic/Latinx participants (7/23, 30%) [40,46,48,49,58,60,62] and only two studies (2/23, 9%) included Black American participants [50,53], and one study (1/23, 4%) evaluated a DMHI among Indigenous communities [35], highlighting ongoing underrepresentation of these groups in culturally adapted digital mental health research, and one study was conducted on Japanese students [55].

### Recruitment Settings

Among the 23 included studies, most investigated DMHIs among participants residing in urban settings (8/23, 35%), typically located near metropolitan areas [32,35,43,46,49,50,55,58]. Most of the studies (14/23, 61%) often relied on community-based recruitment methods such as advertisements, mailing lists, and outreach through community centers [32,35,43,44,46,48,50,53-55,57,58,61,62]. Internet-based recruitment was the second most common strategy, used in 22% (5/23) of studies, primarily through platforms such as social media [44,54,55,57,60]. Seven studies (7/23, 30%) recruited participants directly from outpatient clinical settings located in urban areas [32-35,43,46,53]. A notable proportion of studies (7/23, 30%) recruited through universities and online platforms—to enhance sample diversity and reach [32,34,47,48,53,54,62]. For six studies (6/23, 26%), recruitment settings were not clearly reported, although some recruitment strategies (eg, convenience or snowball sampling) were described [45,47,50,51,56,61].

### Engagement Metrics: Dropout, Adherence, and Attrition Rates

Across the 23 included studies, participant engagement varied substantially. Attrition rates—defined as loss to follow-up—ranged from 5.3% to 87%, with a median attrition rate of approximately 18.4% among studies reporting this outcome. While some studies demonstrated relatively low attrition (eg, <15%) [46,47,56,57,61], some reported notably high rates (>35%) [44,60,62], and five studies did not state attrition rates [33,34,43,54,58], limiting comprehensive comparison. Attrition rates were reported in 61% (14/23) of studies. Dropout rates, reflecting noncompletion of the intervention, also varied widely, from 0% to 66%, with a median dropout rate of 18.7%. Dropout rates were reported in 17 studies (17/23). Adherence rates, or the proportion of sessions or modules completed by participants, ranged from 26.3% to 100%, with a median adherence rate of approximately 71% in studies that reported these data. Adherence rates were reported in 61% (14/23) of studies. High adherence was reported in programs such as internet-delivered cognitive behavioral therapy (Sleep Healthy Using the Internet for Black women) [50], where over 60% of participants completed all modules. However, 39% (9/23) of studies did not report adherence rates [43,48,51,53-58]

### Cultural Adaptation Strategies

Across the included studies, a wide range of cultural adaptation strategies were employed to enhance the relevance and effectiveness of DMHIs for diverse populations. One of the forms of cultural adaptation used in the studies was language translation, implemented in 57% (13/23) of studies to improve linguistic accessibility [32,33,44,46-48,50,51,54,56-58,60]. The other forms of cultural adaptation included content and imagery adaptations that were mainly used in 70% (16/23) of studies to align with cultural norms, such as visuals and metaphors tailored for specific populations [33,35,44,46-48,50,51,53,54,56-58,60-62]. In 70% (16/23) of studies, cultural values and local practices were integrated into the intervention design, including the incorporation of traditional healing methods for Indigenous groups [33,35,44,46-48,50,51,53,54,56-58,60-62]. Stakeholder involvement—including collaboration with cultural experts, local communities, and leaders—was reported in 48% (11/23) of studies [33,35,44,46-48,50,51,53,54,56-58,60-62]. Iterative feedback and refinement processes—using focus groups, cognitive interviews, and pilot trials—were used in 43% (10/23) of studies to adjust the interventions based on user responses [33,35,44,46-48,50,51,53,54,56-58,60-62]. Only three studies (3/23, 13%) employed the Ecological Validity Framework (EVF), guiding systematic adaptation across multiple cultural dimensions [51,56,61]. Similarly, surface- and deep-structure adaptations—which modify both observable aspects like language and deeper cultural constructs—were applied in 9% (2/23) of studies [44,58]. Technology adaptation to locally preferred platforms (eg, WeChat in China) was reported in 35% (8/23) of studies [32,33,47,56-58,60,61]. However, only one study (1/23; 4%) included cultural competency training for providers to ensure culturally sensitive delivery [53].

### Clinical Outcomes

The studies included in this systematic review reported various clinical outcomes, focusing on improvements in mental health symptoms, quality of life, and other relevant measures. Most commonly, the studies targeted insomnia and sleep-related issues as primary clinical outcomes (4/23, 17%) [32,50,54,62], followed by depression (12/23, 52%) [32-34,46-49,54,57,60-62] and anxiety (8/23, 35%) [32,45,47-49,54,56,61,62]. Other notable outcomes included significant reductions in pregnancy-related anxiety among pregnant women in China using a digital guided self-help mindfulness-based intervention [47] an automated bilingual digital health tool in the United States significantly reduced binge drinking episodes [49].

### Methodological Quality Assessment

Overall, most studies (21/23, 91%) were of high methodological quality. Overall, most studies demonstrated clear research aims and employed study designs that were appropriate and well justified in relation to their objectives. The target populations were clearly defined across all studies. Statistical methods were generally well described. Additionally, the key findings of the studies were usually presented clearly, with discussions and conclusions that were largely justified. Most studies also acknowledged their limitations, enhancing transparency. However, improvements are needed in two studies [58,62] by ensuring representative sampling, justifying sample sizes, addressing nonresponse bias, and transparently reporting dropout data.

## Discussion

### Principal Findings

This systematic review synthesized findings from 23 RCTs examining dropout, attrition, and adherence in culturally adapted DMHIs among non-WEIRD adult populations. Participant engagement varied widely, with median dropout and attrition rates around 18% and mean adherence of 71%. Interventions using deep, participatory forms of cultural adaptation—combining translation with locally meaningful content, stakeholder involvement, and iterative refinement—showed the highest adherence (often >75%) and lowest dropout (typically <11%). In contrast, interventions limited to surface-level adaptations such as language translation alone frequently exhibited higher dropout (up to 56%) and lower adherence.

### Patterns in Engagement

Dropout rates ranged from 6% to 87% and appeared to vary depending on adaptation depth [47,60]. Studies integrating multiple culturally grounded elements (eg, language, imagery, values, and delivery context) reported greater retention and engagement. For instance, Zhang et al [32] integrated culturally specific sleep concepts into a CBT-I intervention, achieving a dropout rate of only 6.09%, while Silva et al [40] used culturally resonant telenovela-style content and reported dropout at 8.4%. These findings indicate that culturally resonant content may be linked to greater trust, relevance, and sustained participation.

### Impact of Adaptation Depth

By contrast, interventions that employed surface-level adaptations—such as translation without deeper contextual integration—or lacked explicit cultural adaptation tended to show higher dropout. Spanhel et al [62], for example, provided a non-adapted English CBT intervention to a diverse population and observed a dropout rate of 56%. Similarly, Zeng et al [33] and Guo et al [34] implemented basic linguistic and platform-level adaptations but reported dropout rates of 49.2% and 41%, respectively. These findings suggest that surface-level efforts were typically associated with lower sustained engagement in culturally diverse populations.

### Participatory Design and Implementation

The role of participatory design processes emerged as another important determinant of adherence. Studies like Zhou et al [50] and Lindegaard et al [39] used stakeholder input such as including collaboration with cultural experts, local communities, and leaders and iterative design such as using focus groups, cognitive interviews, and pilot trials, which corresponded to relatively low dropout rates (10.5% and 28%, respectively). However, participatory adaptation alone did not guarantee low attrition, as seen in Barrera et al [60], where despite iterative feedback mechanisms, dropout peaked at 86.97%, possibly due to high geographic and contextual diversity or technological barriers. This highlights the need to complement participatory design with context-sensitive implementation strategies.

### Engagement and Clinical Outcomes

An overall pattern emerged in which studies with lower dropout rates were often observed alongside stronger clinical outcomes. For instance, Zhang et al [32], Silva et al [40], and Sun et al [57] demonstrated both high retention and significant reductions in insomnia, depression, or anxiety. However, some studies with moderate to high dropout (eg, Refs [44,48]) still reported clinical improvements among completers, indicating that while adaptation enhances effectiveness, it may not be sufficient to retain all users without additional strategies to address barriers to access and sustained use.

Despite engagement data from 23 RCTs, a meta-analysis was not possible due to heterogeneity in interventions, populations, outcomes, and definitions of engagement, as well as limited extractable data (≈60%) and small moderator subgroups. We therefore share the descriptive synthesis, and future work using standardized metrics may allow meta-regression.

### Strengths and Limitations of Current Evidence

The reviewed studies highlight several strengths of culturally adapted interventions in supporting engagement and clinical outcomes. Many adapted programs demonstrated higher completion and retention rates, such as tailored versions of Sleep Healthy Using the Internet for Black women [50] and Project Using Practice and Learning to Increase Favorable Thoughts for Hispanic adults [46]. Several interventions also reported improved clinical outcomes, including reduced substance use, greater abstinence, and enhanced sleep or mood symptoms. These positive patterns were most often observed in studies incorporating language congruence and cultural values such as communal participation and sensitivity to race-based stressors.

Despite these successes, several limitations emerged. Many pilot trials had small samples, limiting generalizability and highlighting the need for larger, multi-center validation [32,39,46,53,54]. Perceived cultural relevance also varied within target groups; for instance, in [53], although 84% of participants found the adaptation relevant, some viewed it as “overdone,” reflecting within-group diversity and the importance of facilitator racial matching [53]. Some culturally adapted DMHIs faced challenges in sustaining engagement, such as the online mindfulness course for Pakistani students with high attrition [44] and the Egyptian post-traumatic stress disorder intervention whose participants desired more “human” interaction [56]. A further limitation is that a quantitative meta-analysis or meta-regression was not performed. Considerable heterogeneity in study design, intervention type, and outcome measures—along with inconsistent definitions of adherence and dropout and limited extractable numerical data—made statistical aggregation inappropriate. Subgroup counts were also too small for stable moderator modeling. Moreover, standardized mean differences or confidence intervals for clinical outcomes could not be reported, as most studies used heterogeneous measures and lacked sufficient statistical detail. Consequently, clinical outcomes were synthesized narratively to reflect overall improvement of trends across interventions. Future research should standardize engagement metrics and reporting to enable robust meta-analytic and meta-regression approaches that can better quantify determinants of adherence and attrition. Finally, many studies relied solely on self-reported outcomes and unblinded data collection, increasing the risk of bias [53,56]

### Conclusion

Drawing from these implications, several key recommendations emerge for future research and practice. First, it is essential to prioritize comprehensive cultural adaptation, moving beyond superficial changes to genuinely embed content and delivery methods within the target culture’s values and sociocultural realities [53]. This includes ensuring language congruence [46] and actively involving community members and cultural experts in the design process to ensure adaptations are relevant and address within-group heterogeneity [53]. Second, to support [22] engagement and retention, hybrid models integrating human support should be considered, as noted by users of a culturally adapted web-based post-traumatic stress disorder intervention for Egyptians who desired more “human” interaction and personalization [56]. Proactive monitoring of engagement metrics is also vital to enable timely re-engagement strategies [33]. Third, given that the perceived cultural relevance can be influenced by the race of the intervention facilitator [53], comprehensive cultural competence and implicit bias training for facilitators are recommended to build trust and address potential microaggressions [53].

## Supplementary material

10.2196/80624 Multimedia Appendix 1 Complete search strategy used for PsycINFO, PubMed, and ScienceDirect databases.

10.2196/80624 Multimedia Appendix 2 Detailed study descriptions (Table 1) of included randomized controlled trials.

10.2196/80624 Checklist 1 PRISMA (Preferred Reporting Items for Systematic Reviews and Meta-Analyses) checklist.

## Abbreviations

DMHI: digital mental health intervention
PRISMA: Preferred Reporting Items for Systematic Reviews and Meta-Analyses
PROSPERO: Prospective Specific Evaluation of Reviews
RCT: randomized controlled trial
WEIRD: western, educated, industrialized, rich, democratic
