# Supplementary material for: Retention and Engagement in Culturally Adapted Digital Mental Health Interventions: Systematic Review of Dropout, Attrition, and Adherence in Non-Western, Educated, Industrialized, Rich, Democratic Settings
Source: JMIR Ment Health. 2026 Jan 28;13:e80624. doi: 10.2196/80624 (PMC12850045; doi:10.2196/80624)
Supplement: Multimedia Appendix 1 [file mental-v13-e80624-s001.docx]

**Search Strategy**

(Telemedicine) ) OR (e-health)) OR (e-mental health)) OR (digital mental health)) OR (internet intervention)) OR (web-based intervention)) OR (m-health)) AND (mhealth)) OR (ehealth)) OR (mobile app)) OR (smartphone)) OR (phone app)) OR (cell phone)) AND (adapt)) OR (culturally adapt)) OR (culturally appropriate)) OR (culturally adapted)) OR (culturally responsive)) OR (culturally tailored) Filters: in the last 10 years.
